# Healthy Timing and Spacing of Pregnancy: Reducing Mortality Among Women and Their Children

**DOI:** 10.9745/GHSP-D-19-00262

**Published:** 2019-08-22

**Authors:** Ellen Starbird, Kathryn Crawford

**Affiliations:** a Office of Population and Reproductive Health, United States Agency for International Development (USAID), Washington, DC, USA.; b Maternal and Child Health and Nutrition Office, United States Agency for International Development (USAID), Washington, DC, USA.

## Abstract

Accessible, affordable, and high-quality postabortion care (PAC) can prevent maternal death and disability and provides an important opportunity to prevent future unintended pregnancies. This supplement offers learnings on PAC provision from the community of partners around the world, including service delivery and community engagement models, approaches to support facility-based providers, best practices in pre- and post-procedure counseling, and approaches to institutionalize PAC in public- and private-sector health systems.

Research from around the world shows that the length of the interval between a woman’s birth and her next pregnancy directly relates to the risk of infant, child, and maternal mortality.^1^^–^^3^ In summary, the shorter the birth-to-pregnancy interval, the higher the risk to women and their children. In addition, pregnancies that are too closely spaced or that occur among adolescents younger than 18 also carry with them a higher risk of preterm birth and low birth weight for infants, as well as maternal pregnancy- and birth-related complications, such as anemia and obstetric fistula.

Three evidence-based global recommendations for healthy timing and spacing of pregnancy can lead to significantly improved maternal and child health outcomes:

- Women should **delay their first pregnancy until at least age 18**.
- After a live birth, women should wait at least **24 months** before attempting the next pregnancy to reduce health risks for the mother and the baby.
- After a miscarriage or induced abortion, women should wait at least **6 months** before attempting the next pregnancy to reduce health risks for the mother and baby.

## SYNERGIES BETWEEN SUPPORT FOR POSTABORTION FAMILY PLANNING AND POSTPARTUM FAMILY PLANNING

Over much of the same period that the United States Agency for International Development (USAID) has supported postabortion care (PAC) programs, USAID has also supported efforts to strengthen voluntary postpartum family planning by better understanding women’s needs and current practices in the extended postpartum period; raising awareness about postpartum fertility, particularly in relation to exclusive breastfeeding and the Lactational Amenorrhea Method; expanding the range of contraceptive methods available for postpartum women; and increasing the opportunities to offer these to postpartum women during the continuum of care for mothers, newborns, and children. Given the many parallel investments to strengthen postabortion and postpartum family planning service delivery and demand generation (e.g., provider training, organization of integrated services, supply chain and contraceptive commodities, recordkeeping, client counseling, social and behavior change at community and interpersonal levels, and more), USAID sees both of these as fundamental opportunities for integrated programming to address the lifesaving health care and contraceptive needs of women at key points during their reproductive life course.

## USAID’S SUPPORT FOR POSTABORTION CARE: SAVING WOMEN’S LIVES

PAC is a lifesaving intervention. When it is accessible, affordable, of high quality, and performed by capable health care providers, PAC can prevent maternal death and disability. PAC is an integrated service delivery model that combines emergency and preventive services. It also combines both maternal health services (emergency treatment) and family planning services (voluntary counseling and service delivery) and is completed **before** the client is discharged from the facility. Emergency treatment is provided immediately, based on country protocols, for complications from miscarriage or induced abortion and considered a key component of emergency obstetric care. Even if a woman wants to become pregnant again, most guidance suggests waiting 6 months before attempting another pregnancy to reduce the potential for low birth weight, premature birth, and maternal anemia.^4^ Research demonstrates that when clients are routinely counseled and offered voluntary contraception as part of PAC, most will opt to leave the facility with a family planning method.^5^^,^^6^

Since 1994, USAID has supported implementation of PAC programs in more than 40 countries to address complications related to miscarriage and incomplete abortion.^7^ USAID-supported PAC programs comprise emergency treatment for complications of induced or spontaneous abortion, counseling on and provision of family planning options, and community mobilization (Figure). These programs do not perform or actively promote abortion as a method of family planning. Because PAC clients can become pregnant almost immediately after abortion, offering voluntary family planning counseling and services is an important opportunity to prevent future unintended pregnancies.

**FIGURE:**
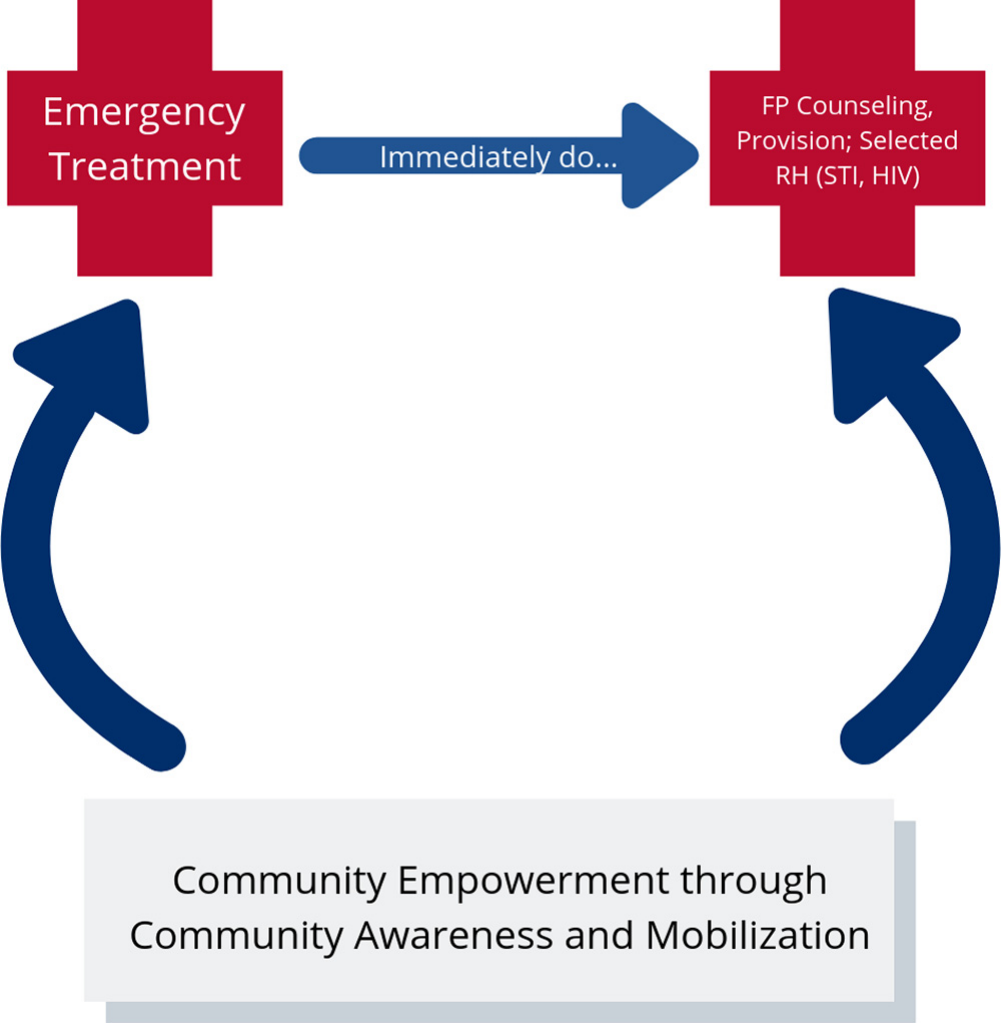
USAID’s Postabortion Care Model Abbreviations: FP, family planning; RH, reproductive health; STI, sexually transmitted infection; USAID, United States Agency for International Development.

USAID’s PAC programs have the following 3 components:

1. **Emergency treatment:** prompt management of potentially life-threatening complications including hemorrhage, infection, injury to internal organs, and shock. Treatment for postabortion complications often occurs in the same location where deliveries are performed by skilled birth attendants. Programs follow country guidelines regarding how to safely treat incomplete abortion. Clients will receive counseling pre- and post-procedure on what to expect during and after treatment, how to recognize danger signs, and when and where to seek additional care. Programs should support providers to provide respectful, dignified care throughout, including pain management.
2. **Family planning counseling and service delivery:** includes pre- and post-procedure client-centered counseling on when clients can experience a return to fertility after PAC, reproductive intentions, and healthy timing of pregnancy. If a client opts for a family planning method, she is offered a choice of a range of voluntary contraceptive methods. Although PAC clients can use any of a wide range of contraceptive methods (Box), some are more appropriate for immediate use than others and the timing depends on the method, the woman’s condition, and the method of her treatment for complications (medical or surgical). Where feasible, sexually transmitted infection evaluation and treatment and HIV counseling and/or referral for HIV testing are also provided as needed. Support to clients who have experienced gender-based violence may also be offered or referred, where feasible.
  BOX **Contraceptive Methods for Postabortion Care**
  **Can start immediately:**
  - Hormonal methods: implants, monthly injectables, injectables, combined oral contraceptive pills, progestin-only pills, progestin-only injectables, combined patch, emergency contraceptive pills.
  - Barrier methods: male or female condoms.
  - Intrauterine devices (IUDs): copper-bearing or levonorgestrel-releasing IUDs can be provided immediately after emergency treatment of complications if there is no infection or when infection is ruled out and resolved and any injury has healed. However, IUD insertion after medical treatment of emergency complications requires the client to return for a follow-up visit.
  - Diaphragms, cervical caps, and combined vaginal ring: can be offered once injury is ruled out or after any injury to the genital tract has healed.
  - Permanent methods: tubal ligation or vasectomy (for her partner). Permanent methods can be offered after the client has had time to rest and recover from any sedation and is not stressed or in pain. Counsel carefully, ensure informed consent, and be sure to mention available reversible methods.
  **Delay use:**
  - Fertility awareness methods: Standard Days Method or TwoDay Method. It is recommended that women start these methods after their regular menstrual pattern returns.
  Source: World Health Organization, 2018.^4^
3. **Community empowerment through community awareness and mobilization:** application of the community action cycle to raise awareness about complications from miscarriage and induced abortion, facilitate connections with local health services, and empower communities to demand quality, effective PAC.

## POLICY AND LEGISLATIVE REQUIREMENTS GOVERNING USAID’S POSTABORTION CARE PROGRAMMING

USAID-funded PAC programs are guided by several statutory and policy requirements that include restrictions related to abortion. Additionally, the principles of voluntarism and informed choice articulated in legislative and policy requirements guide USAID’s family planning program, including PAC. Programming for PAC is permitted under USAID’s statutory and policy restrictions related to abortion. More specifically, PAC is explicitly permitted in the standard provision that implements the Protecting Life in Global Health Assistance policy (formerly the Mexico City Policy).^8^^,^^9^

## DOCUMENTING AND SHARING EXPERIENCES IN POSTABORTION CARE

This special supplement is an opportunity to learn from the community of partners around the world working to respond to women’s needs by providing lifesaving PAC. As we strive to make these services accessible to women experiencing complications from miscarriage or induced abortion, it is important that we document and disseminate our experiences so we can learn the following:

- Service delivery models and approaches for how to best provide lifesaving treatment and client-centered care with available health care cadres and facilities, where women are, in all types of country settings, from urban to rural, from humanitarian to development
- Approaches to support facility-based providers through competency-based training and supportive supervision and adequate equipment and supplies
- Best practices in pre- and post-procedure counseling, including voluntary family planning, to avoid future unintended pregnancies
- Community engagement models to promote care-seeking behavior, reduce stigma, and support women experiencing obstetric emergencies
- Effective approaches to institutionalize PAC in public- and private-sector health systems to support countries in their ability to offer this important, lifesaving health service beyond donor assistance

The open-access information in each of the articles included in this supplement provides the details of how PAC programs are implemented in addition to the results of these programs, with the ultimate goal of helping those who design, implement, manage, evaluate, and otherwise support health programs to more easily adopt and adapt the strategies and approaches.
